# Water Extract of *Curcuma longa* L. Ameliorates Non-Alcoholic Fatty Liver Disease

**DOI:** 10.3390/nu11102536

**Published:** 2019-10-21

**Authors:** Jeongeun Mun, Shintae Kim, Ho-Geun Yoon, Yanghee You, Ok-Kyung Kim, Kyung-Chul Choi, Yoo-Hyun Lee, Jeongmin Lee, Jeongjin Park, Woojin Jun

**Affiliations:** 1 Division of Food and Nutrition, Chonnam National University, Gwangju 61186, Korea; mjo3214@naver.com (J.M.); rotoman1@naver.com (S.K.); yewha@naver.com (Y.Y.); 20woskxm@chonnam.ac.kr (O.-K.K.); 2 Department of Biochemistry and Molecular Biology, College of Medicine, Yonsei University, Seoul 03722, Korea; yhgeun@yuhs.ac.kr; 3 Department of Biomedical Sciences, University of Ulsan College of Medicine, Seoul 05505, Korea; choikc75@amc.seoul.kr; 4 Department of Food and Nutrition, University of Suwon, Gyeonggido 18323, Korea; creamut@suwon.ac.kr; 5 Department of Medical Nutrition, Kyung Hee University, Gyeonggido 17104, Korea; jlee2007@khu.ac.kr

**Keywords:** *Curcuma longa* L., non-alcoholic fatty liver disease, fatty acid uptake, lipid accumulation, oxidative stress

## Abstract

Our aim was to investigate whether hot water extract (CLW) of *Curcuma longa* L. could prevent non-alcoholic fatty liver disease (NAFLD). HepG2 cells were treated with free fatty acid (FFA) mixture (oleic acid: palmitic acid, 2:1) for 24 h to stimulate in vitro fatty liver. In addition, C57BL/6 mice were fed 60 kcal% high-fat (HF) diet for eight weeks to induce fatty liver in vivo. Intracellular reactive oxygen species (ROS) and malondialdehyde (MDA) productions were increased by FFA and HF-diet, but supplementation with CLW significantly decreased these levels. CLW treatment ameliorated antioxidant activities that were suppressed by exposure to the FFA and HF-diet. Cluster of differentiation 36 (CD36) and fatty acid transport proteins (FATP2 and FATP5) were increased in HF-diet groups, while CLW suppressed their expression levels. Moreover, sterol regulatory element-binding protein-1c (SREBP-1c), acetyl-coenzyme A carboxylase (ACC), and fatty acid synthase (FAS) expression levels were down-regulated in the CLW groups compared to HF-diet groups. On the other hand, 5′ adenosine monophosphate-activated protein kinase (AMPK), Peroxisome proliferator-activated receptor alpha (PPAR-α), and carnitine palmitoyltransferase 1 (CPT-1) expressions were up-regulated in the CLW groups. HF-diet fed mice showed high hepatic triglycerides (TG) content compared to the normal diet mice. However, the administration of CLW restored the hepatic TG level, indicating an inhibitory effect against lipid accumulation by CLW. These results suggest that CLW could be a potentially useful agent for the prevention of NAFLD through modulating fatty acid uptake.

## 1. Introduction

The liver is the central organ of lipid metabolism. Dietary fatty acids are transported to the liver and oxidized to produce as much energy as the hepatocyte needs. The excess fatty acids are converted to triglycerides (TG) and stored in the hepatocyte [1]. When the amount of TG accounts for more than 5% of the liver weight, the organ function is impaired, and this condition is known as fatty liver disease. Chronic alcohol intake has long been considered the major cause of alcoholic fatty liver, while non-alcoholic fatty liver has been shown to be unrelated to alcohol intake [2]. The causes of non-alcoholic fatty liver are lifestyle habits, which include excessive dietary intake and lack of energy consumption [3]. Therefore, it is often accompanied by lifestyle diseases such as obesity, diabetes, and metabolic syndrome. In recent times, with the growing incidence of these diseases, the attention paid to non-alcoholic fatty liver is gradually increasing [4]. Non-alcoholic fatty liver disease (NAFLD) shows various stages. Simple hepatic steatosis is characterized by lipid accumulation in hepatocytes. Non-alcoholic steatohepatitis (NASH) can be further developed into cirrhosis and hepatocellular carcinoma. Although many factors such as obesity, diabetes, and inflammation have been implicated in relation to NAFLD in humans [5,6], the underlying mechanism of the development and progression of NAFLD remains unclear.

When free fatty acid (FFA) is overloaded in the body, FFA uptake factors such as cluster of differentiation 36 (CD36) and fatty acid transport proteins (FATPs) are significantly up-regulated, and then excessive FFA is accepted into the liver, gradually accumulating [7]. According to previous studies, palmitic acid and high-fat (HF) diet markedly increased CD36 expression, which is closely associated with the development of NAFLD [8,9]. Furthermore, several studies have indicated that fatty acid overflow by activated CD36 subsequently produced a higher level of reactive oxygen species (ROS), which could be linked to oxidative stress [10].

Oxidative stress is the imbalance between the production of ROS and the activation of antioxidants in the body and results in several metabolic disorders [11]. It particularly produces a disturbance in lipid metabolism, eventually leading to lipid accumulation in hepatocytes. Of the several existing pathogenic mechanisms of NAFLD, oxidative stress is considered a major contributor. According to a previous study, uric acid-induced oxidative stress in hepatocytes significantly elevated the expression of lipogenesis factors such as acetyl-coenzyme A carboxylase (ACC), fatty acid synthase (FAS), and sterol regulatory element-binding protein-1c (SREBP-1c) [12]. Another study has shown that an HF diet destroyed the antioxidant defense system by decreasing superoxide dismutase (SOD) and catalase (CAT) activities, and markedly increased the production of ROS [13]. To prevent metabolic disorders due to oxidative stress, antioxidative capability that suppresses or eliminates ROS production plays an important role in the body. Plants contain a variety of phytochemicals that have antioxidant ability to effectively remove ROS [14].

*Curcuma longa* L. is a flowering plant of the ginger family (Zingiberaceae). As *C. longa* possesses natural antioxidants, which leads to free radical scavenging potential, it has been widely used to prevent many diseases, including liver injury [15]. 

The aim of our study was to investigate in vitro and in vivo protective effects of hot water extract (CLW) from *C. longa* on the development of fatty liver. Furthermore, the mode of action against lipid accumulation and oxidative stress in non-alcoholic liver damage was determined.

## 2. Materials and Methods

### 2.1. Sample and Chemicals

*C. longa*, which was supplied by the SDC R&D Center (Damyang, Korea), was extracted with 20 volumes of hot water at 250 °C for 3 h. The extracted solution was filtered and concentrated using an evaporator under vacuum conditions. The concentrate was lyophilized and stored at -20°C until further use. Minimum essential medium (MEM), fetal bovine serum (FBS), antibiotics, trypsin-ethylenediamine tetraacetic acid, and Hank’s balanced salt solution were products of Gibco BRL (Grand Island, NY, USA). Sodium salt of 2,3-bis[2-methoxy-4-nitro-5-sulfophenyl]-2H-tetrazolium-5-carboxyanilide inner salt or XTT, H_2_O_2_, 4-nitrocatechol, 2’,7’-dichlorofluorescein diacetate (DCF-DA), sodium oleate, sodium palmitate, and Oil Red O were purchased from Sigma-Aldrich (St. Louis, MO, USA). All other chemicals were of analytical reagent grade.

### 2.2. Cell Culture

Human hepatocellular carcinoma cell line HepG2 cells were obtained from the American Type Culture Collection (Manassas, VA, USA). The cells were cultured in MEM supplemented with 10% FBS and 1% penicillin/streptomycin and maintained in 10 cm dishes at 37 °C under a humidified atmosphere of 5% CO_2_.

### 2.3. FFA-Induced Lipid Overloading in HepG2 Cells

HepG2 cells were seeded at 1 × 10^5^ cells/well in a 24-well culture plate and incubated for 24 h to induce excessive lipid accumulation in an in vitro hepatic steatosis model. On day 2, when the cells reached approximately 80% confluency, they were pretreated with CLW solution for 2 h, and then, 1 mM FFA mixture was added into each well. To prepare the FFA mixture, sodium oleate and sodium palmitate were conjugated with a culture medium containing 1% bovine serum albumin (BSA). The FFA mixture (2:1 ratio of oleate: palmitate) was diluted in the culture medium to obtain the desired final concentration. Control cells were treated with 1% BSA only.

### 2.4. Measurement of Intracellular ROS Formation

Intracellular ROS levels were detected using the fluorescence probe (DCF-DA). After 24 h incubation of the HepG2 cells that had been seeded in a 24-well plate at a concentration of 1 × 10^5^ cells/well, the cells were treated with 50 and 100 μg/mL CLW and 1 mM FFA mixture, respectively, for 2 h. The cells were then exposed to 30 μM DCF-DA for 30 min at 37°C. Fluorescence intensity of the cells was measured using a fluorescence microplate reader (BioTek Instruments, Winooski, VT, USA) with an excitation wavelength of 485 nm and an emission wavelength of 530 nm. Control cells were treated with 1% BSA only.

### 2.5. Measurement of Lipid Accumulation (Oil Red O Staining)

To evaluate the accumulation of intracellular neutral lipids levels, HepG2 cells were treated with 50 and 100 μg/mL CLW and 1 mM FFA mixture for 24 h, respectively. Then, the cells were fixed with 10% formalin for 30 min, followed by staining with Oil Red O solution for 40 min. To remove the remnants of the Oil Red O reagent, the stained cells were washed 3 times with phosphate-buffered saline (PBS) and then observed under a microscope (Leica DMi1; Leica Microsystems, Wetzlar, Germany). Lipid droplets were extracted using 60% isopropanol and assessed colorimetrically at 510 nm. Control cells were treated with 1% BSA only. 

### 2.6. Animal Experiments

Seven-week-old male C57BL/6 mice were purchased from Orient Bio (Seongnam, Korea). They were acclimated for 1 week before experiments. The animals were housed in stainless steel cages under controlled laboratory conditions (20–25 °C, 55%–60% humidity, and 12 h light/dark cycle of lighting). The Institutional Animal Care and Use Committee of Chonnam National University approved the protocol for the animal study. Animals were cared for according to the “Guidelines for Animal Experiments” established by Chonnam National University (CNU IACUC-YB-2018-55). For the experiments, the mice were randomly divided into 5 groups (*n =* 10 per group) as follows; (1) CON: Mice fed a normal diet (AIN-76A diet, Research Diet, Inc., New Brunswick, NJ, USA); (2) HF: Mice fed a 60 kcal% fat diet (D12492, Research Diet, Inc.); (3) C-LOW: Mice fed high-fat diet plus CLW 300 mg/kg/b.w./day; (4) C-HIGH: Mice fed high-fat diet plus CLW 900 mg/kg/b.w./day; and (5) SILY: Mice fed high-fat diet plus silymarin 50 mg/kg/b.w./day. The animals were fed ad libitum for 8 weeks. Blood samples were collected from the postcaval vein of each animal immediately after death and centrifuged at 3000 rpm for 15 min. The serum was stored at -80 °C until further analysis. Each tissue was immediately weighed and stored at -80°C until further analysis.

### 2.7. Assays for Serum Marker Enzymes, and Hepatic Triglyceride, and Total Cholesterol

Aspartate aminotransferase (AST) and alanine aminotransferase (ALT) were measured using assay kits (Asan Pharmaceutical, Seoul, Korea). For analyses of hepatic TG and total cholesterol, the frozen liver tissue was homogenized in a glass-Teflon homogenizer (Daihan, Korea) with PBS and centrifuged at 13,000 rpm for 30 min. The liver lipids were extracted by the Folch method [16] and quantified. Hepatic TG and total cholesterol (TC) levels were measured by assay kits (Asan Pharm).

### 2.8. Hematoxylin and Eosin Staining

For hematoxylin and eosin (H&E) staining, the liver was fixed in 10% formaldehyde, embedded in paraffin, and cut into 10 µm sections. The sections were stained with H&E and then observed under a microscope.

### 2.9. Measurement of Antioxidant Enzyme Activity

For the antioxidant activity assays, the liver was homogenized in PBS. The suspension was centrifuged at 13,000× *g* for 15 min at 4 °C, and the supernatant was used for the assay. The amount of protein was estimated using the Bradford assay. The SOD activity was measured by the method of Alam [17], and CAT activity was assayed by the method of Garg [18]. The activities of hepatic glutathione-*S*-transferase (GST), GPx, and GR were determined by methods of Molina [19], Al Batran [20], and Calberg and Mannervik [21], respectively. Reduced glutathione (GSH) level was measured using the method of Akerboom and Sies [22]. To measure the lipid peroxidation, the concentration of malondialdehyde (MDA) was monitored with thiobarbituric acid reactive substance formation as described by Draper and Hadley [23], and calculated using a standard curve.

### 2.10. Total RNA Isolation and Real-Time Polymerase Chain Reaction

Total RNA was extracted from the liver by an easy-BLUE^TM^ total RNA extraction kit (Intron Biotechnology, Sungnam, Korea) according to the manufacturer’s instructions. Nano-drop measurements of RNA samples were processed to determine the quality and quantity of the RNA. cDNA was synthesized from the purified total RNA using an iScript™ cDNA synthesis kit (Bio-Rad Laboratories, Hercules, CA, USA). Real-time polymerase chain reaction (RT-PCR) was performed using an SYBR green RT-PCR kit and custom-designed primers (Table 1) with the CFX96 Touch^TM^ real-time PCR detection system (Bio-Rad). The cDNA was amplified for 40 cycles of denaturation (95 °C for 30 s), annealing (58 °C for 30 s), and extension (72 °C for 45 s).

### 2.11. Western Blotting

Equal amounts of proteins (100 μg/lane) from the liver were separated by sodium dodecyl sulfate-polyacrylamide gel electrophoresis and transferred to polyvinylidene difluoride membranes (Bio-Rad). The membranes were blocked with blocking buffer (10% blocker BSA in PBS, Thermo Fisher Scientific) for 30 min before incubating with the first antibody at 4 °C overnight. Antibodies against AMPK, phospho-AMPK, ACC, phospho-ACC, and β-actin were purchased from Cell Signaling Technology (Danvers, MA, USA). Thereafter, the membranes were incubated with a secondary antibody (anti-rabbit immunoglobulin G (IgG), Cell Signaling Technology) for 2 h. Bands were detected via enhanced chemiluminescence (ECL) using ECL western blotting detection reagents (Bio-Rad).

### 2.12. Statistical Analysis

All data are presented as mean ± S.E. Data were statistically evaluated via one-way analysis of variance (ANOVA) using SPSS statistical procedures for Windows (SPSS PASW Statistics 22.0, SPSS Inc. Chicago, IL, USA), and Duncan’s multiple range test was used to compare significant differences between groups (*p* < 0.05).

## 3. Results

### 3.1. Effect of CLW on Antioxidant Activity in FFA-Treated Cells

**Figure 1:**
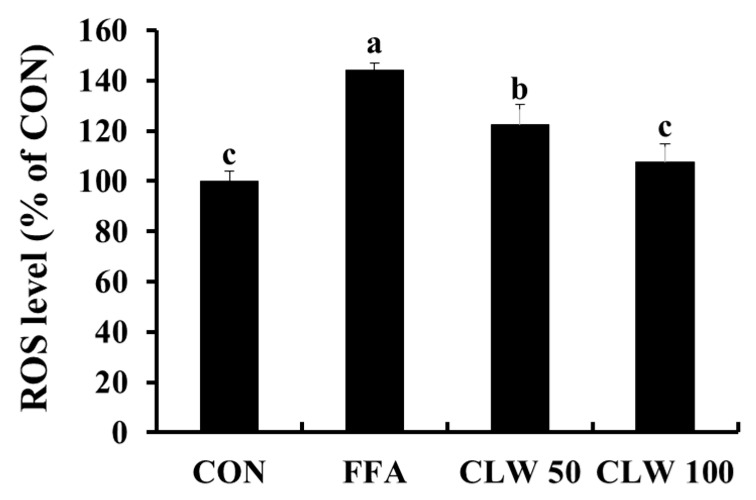
Effect of water extract (CLW) from *Curcuma longa* L. on the formation of intracellular reactive oxygen species (ROS) in free fatty acid-treated HepG2 cells. CON, normal control group; FFA, free fatty acid-treated group; CLW50 and CLW100, pretreatment with CLW 50 and 100 μg/mL groups, respectively. Data are expressed as mean ± S.E., and different letters indicate significant differences (*p* < 0.05, a > b > c), as determined via Duncan’s multiple range test.

The changes in intracellular ROS levels by CLW in FFA-treated cells are depicted in Figure 1. Compared to the control group, the intracellular ROS level of only the FFA-treated group was significantly increased. When the cells were pretreated with 50 and 100 μg/mL CLW, intracellular ROS formation was suppressed in a dose-dependent manner compared to that in the only FFA-treated group.

### 3.2. Effect of CLW on Lipid Accumulation in FFA-Treated Cells

**Figure 2:**
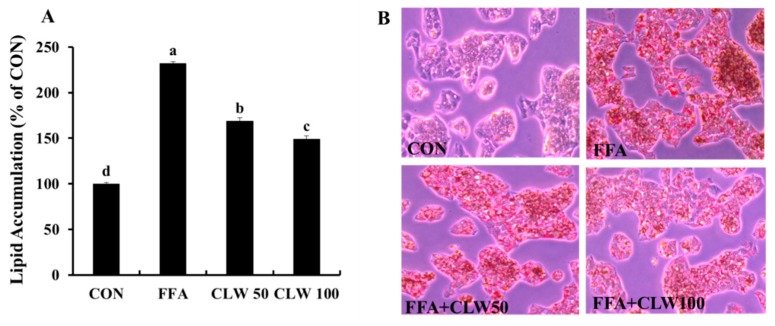
Effect of water extract (CLW) from *Curcuma longa* L. on lipid accumulation in free fatty acid-treated HepG2 cell. (**A**) Intracellular lipid levels were colorimetrically measured at 510 nm. (**B**) The stained cells were observed under a microscope (400×). CON, normal control group; FFA, FFA, free fatty acid-treated group; CLW50 and CLW100, pretreatment with CLW 50 and 100 μg/mL groups, respectively. Data are expressed as mean ± S.E., and different letters indicate significant differences (*p* < 0.05), as determined via Duncan’s multiple range test.

Lipid accumulation had noticeably increased in the FFA-treated group compared to that in the control group, while CLW supplementation decreased lipid accumulation in a dose-dependent manner (Figure 2A). Oil Red O staining image of the FFA-treated group showed more in the cell than the other groups. On the other hand, pretreated groups with CLW had less lipid droplets, indicating a decrease in lipid accumulation (Figure 2B).

### 3.3. Effect of CLW on HF-Induced Hepatotoxicity in Mice

**Figure 3:**
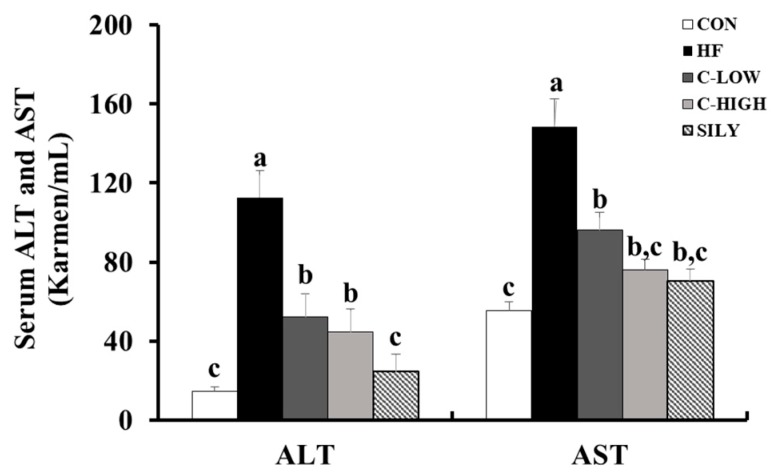
Effect of water extract (CLW) from *Curcuma longa* L. on hepatic markers in the serum of high-fat diet-fed mice. ALT; alanine aminotransferase, AST; aspartate aminotransferase. CON, normal control diet; HF, 60% high-fat diet; C-LOW, 60% high-fat diet plus 300 mg/kg b.w./day of CLW; C-HIGH, 60% high-fat diet plus 900 mg/kg b.w./day of CLW; SILY, 60% high-fat diet plus 50 mg/kg b.w./day of silymarin. Data are expressed as mean ± S.E. (*n* = 10), and different letters indicate significant differences (*p* < 0.05), as determined via Duncan’s multiple range test.

**Figure 4:**
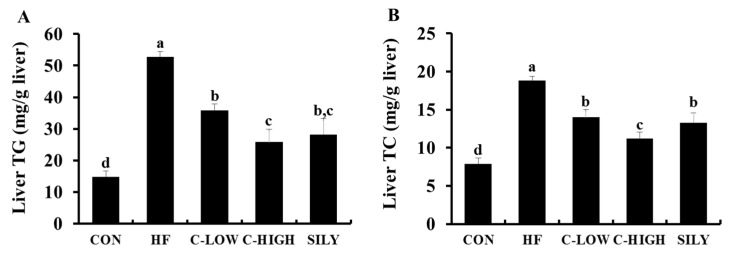
Effect of water extract (CLW) from *Curcuma longa* L. on hepatic triglyceride and total cholesterol in high-fat diet-fed mice. (**A**) TG; triglyceride, (**B**) TC; total cholesterol. CON, normal control diet; HF, 60% high-fat diet; C-LOW, 60% high-fat diet plus 300 mg/kg b.w./day of CLW; C-HIGH, 60% high-fat diet plus 900 mg/kg b.w./day of CLW; SILY, 60% high-fat diet plus 50 mg/kg b.w./day of silymarin. Data are expressed as mean ± S.E. (*n* = 10), and different letters indicate significant differences (*p* < 0.05), as determined via Duncan’s multiple range test.

Serum AST and ALT activities were useful biological indicators of liver damage and hepatotoxicity. These enzyme activities were significantly elevated in mice administered an HF diet. However, CLW co-treatment for eight weeks remarkably decreased the serum levels of these indicators (Figure 3). Notably, supplementation with CLW at a dose of 300 mg/kg b.w./day recovered the impaired liver functions resulting from HF-induced toxicity. As shown in Figure 4, the liver TG and TC levels in the HF group were higher than those in the control group, whereas co-administration of CLW markedly reduced TG and TC levels. These results suggested that CLW supplementation suppressed lipid accumulation in the liver.

### 3.4. Effect of CLW on Histopathological Morphology

**Figure 5:**
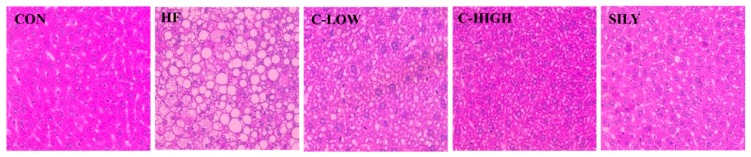
Effect of water extract (CLW) from *Curcuma longa* L. on liver histopathological changes in high-fat diet-fed mice. Liver sections of mice fed a normal control diet or a high-fat diet for 8 weeks, stained with hematoxylin and eosin (H&E; bars: 50 μm). CON, normal control diet; HF, 60% high-fat diet; C-LOW, 60% high-fat diet plus 300 mg/kg b.w./day of CLW; C-HIGH, 60% high-fat diet plus 900 mg/kg b.w./day of CLW; SILY, 60% high-fat diet plus 50 mg/kg b.w./day of silymarin.

The livers of the control mice revealed no abnormal appearance or histopathological change. However, the HF diet caused fatty liver in mice, as demonstrated by outstandingly numerous lipid droplets compared to the control liver (Figure 5). The hepatic lipid droplets induced by the HF diet were significantly reduced by treatment with CLW. When administered CLW at a dose of 900 mg/kg b.w./day, liver conditions were ameliorated to the normal liver tissues.

### 3.5. Changes in Antioxidant Activities in Fatty Liver by CLW Treatment

**Figure 6:**
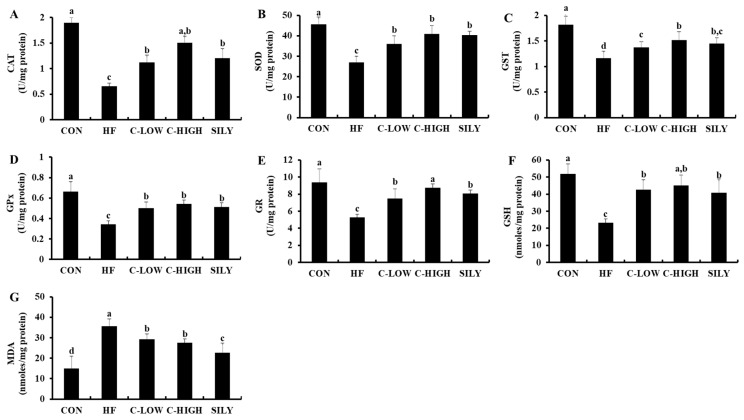
Effect of water extract (CLW) from *Curcuma longa* L. on antioxidant status in high-fat diet-fed mice. (**A**) Catalase (CAT) activity, (**B**) superoxide dismutase (SOD) activity, (**C**) glutathione-*S*-transferase (GST) activity, (**D**) glutathione peroxidase (GPx) activity, (**E**) glutathione reductase (GR) activity, (**F**) reduced glutathione (GSH) level, (**G**) malondialdehyde (MDA) level in the livers of different groups. Data are expressed as mean ± S.E. (*n* = 10), and different letters indicate significant differences (*p* < 0.05) as determined via Duncan’s multiple range test. CON, normal control diet; HF, 60% high-fat diet; C-LOW, 60% high-fat diet plus 300 mg/kg b.w./day of CLW; C-HIGH, 60% high-fat diet plus 900 mg/kg b.w./day of CLW; SILY, 60% high-fat diet plus 50 mg/kg b.w./day of silymarin.

The functions of liver antioxidants in HF diet-fed mice are summarized in Figure 6A–F. In the HF group, levels of CAT, SOD, GST, GPx, GR, and GSH were significantly dropped compared to those of the control group. However, co-treatment with CLW at a dose of 300 mg/kg b.w./day completely prevented a decrease in liver antioxidants. In this study, silymarin, a well-known hepatoprotectant obtained from the milk thistle, was used as a positive control. The antioxidant levels of CLW were similar to those of silymarin. The oxidative stress induced by an HF diet caused a significant increase in MDA levels, a key biomarker of oxidative stress, in comparison with the control group (Figure 6G). On the other hand, supplementation with CLW partially prevented the elevation of MDA. These results showed that CLW suppressed the oxidative stress in the liver.

### 3.6. Effect of CLW on Fatty Acid Uptake-Related mRNA Expression in HF Diet-fed Mice

**Figure 7:**
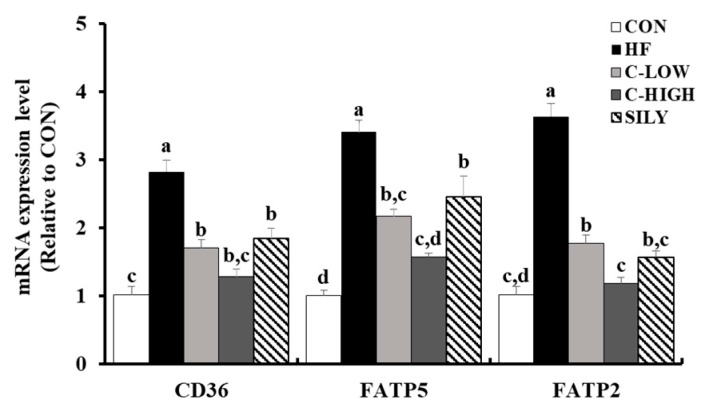
Effect of water extract (CLW) from *Curcuma longa* L. on fatty acid uptake in high-fat diet-fed mice. mRNA expression levels of fatty acid uptake factors, including a cluster of differentiation 36 (CD36), fatty acid transport protein 2 (FATP2), and FATP5. CON, normal control diet; HF, 60% high-fat diet; C-LOW, 60% high-fat diet plus 300 mg/kg b.w./day of CLW; C-HIGH, 60% high-fat diet plus 900 mg/kg b.w./day of CLW; SILY, 60% high-fat diet plus 50 mg/kg b.w./day of silymarin. Data are expressed as mean ± S.E. (*n* = 10), and different letters indicate significant differences (*p* < 0.05), as determined via Duncan’s multiple range test.

Expression levels of mRNA related to fatty acid uptake, such as CD36, FATP5, and FATP2, were up-regulated in the HF group (Figure 7). The HF diet actively induced inflow of free fatty acids in the liver, resulting in fatty liver disease. However, CLW groups showed significantly down-regulated fatty acid levels, which were comparable to those of the SILY group. These results indicated that fatty acid uptake was blocked by CLW supplementation.

### 3.7. Effect of CLW on Lipid Accumulation-Related mRNA Expression in HF Diet-fed Mice

**Figure 8:**
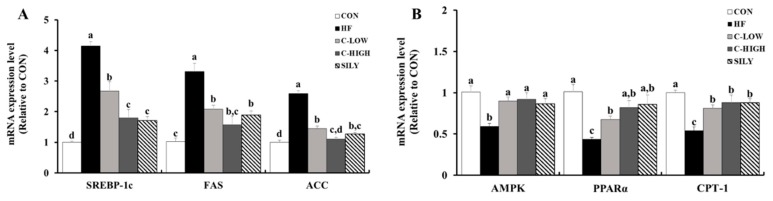
Effect of water extract (CLW) from *Curcuma longa* L. on lipid metabolism in high-fat diet-fed mice. (**A**) mRNA expression levels of fatty acid synthesis factors, including sterol regulatory element-binding protein-1c (SREBP-1c), fatty acid synthase (FAS), and acetyl-coenzyme A carboxylase (ACC). (**B**) mRNA expression levels of β-oxidation factors, including 5′ adenosine monophosphate-activated protein kinase (AMPK), and peroxisome proliferator-activated receptor alpha (PPAR- α), carnitine palmitoyltransferase 1 (CPT-1). CON, normal control diet; HF, 60% high-fat diet; C-LOW, 60% high-fat diet plus 300 mg/kg b.w./day of CLW; C-HIGH, 60% high-fat diet plus 900 mg/kg b.w./day of CLW; SILY, 60% high-fat diet plus 50 mg/kg b.w./day of silymarin. Data are expressed as mean ± S.E. (*n* = 10), and different letters indicate significant differences (*p* < 0.05), as determined via Duncan’s multiple range test.

In comparison with the control mice, HF diet mice showed increased mRNA expression levels related to fatty acid synthesis, such as SREBP-1c, FAS, and ACC by approximately 4.1, 3.3, and 2.6-fold, respectively (Figure 8A). Supplementation with CLW reversed the elevations of mRNA factors involved in fatty acid synthesis. Moreover, mice co-treated with CLW restored the β-oxidation factors such as 5′ adenosine monophosphate-activated protein kinase (AMPK), peroxisome proliferator-activated receptor-α (PPAR-α), and carnitine palmitoyltransferase 1 (CPT-1), indicating CLW as the accelerant of lipid consumption (Figure 8B).

### 3.8. Effect of CLW on Activation of AMPK and ACC in HF Diet-fed Mice

**Figure 9:**
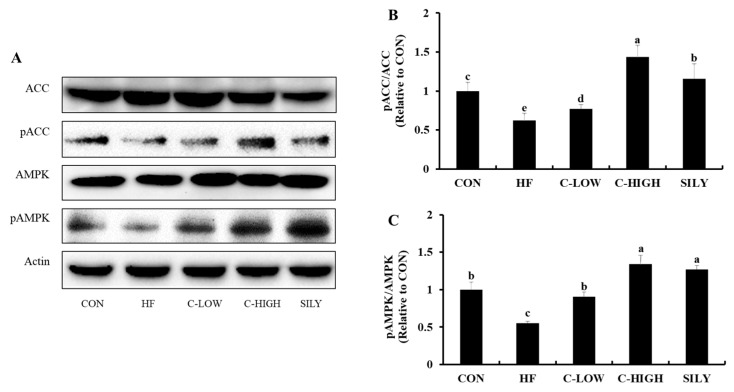
Effect of water extract (CLW) from *Curcuma longa* L. on activation of AMPK and ACC in high-fat diet-fed mice. (**A**) 5′ adenosine monophosphate-activated protein kinase (AMPK) and phosphorylation of acetyl-coenzyme A carboxylase (ACC) protein levels were monitored by Western blot analysis. (**B**) and (**C**) Protein density of ACC and AMPK. CON, normal control diet; HF, 60% high-fat diet; C-LOW, 60% high-fat diet plus 300 mg/kg b.w./day of CLW; C-HIGH, 60% high-fat diet plus 900 mg/kg b.w./day of CLW; SILY, 60% high-fat diet plus 50 mg/kg b.w./day of silymarin. Data are expressed as mean ± S.E. (*n* = 10), and different letters indicate significant differences (*p* < 0.05), as determined via Duncan’s multiple range test.

AMPK and ACC activities in the liver were measured by performing a western blot assay. Both phosphorylated AMPK to AMPK ratio and the phosphorylated ACC to ACC ratio were significantly decreased in HF diet-fed mice compared to the control mice (Figure 9). On the other hand, the CLW administration increased these ratios back to the normal range. These results suggested that CLW might decrease lipid accumulation in the liver via enhancing AMPK activation and suppressing ACC activation. 

## 4. Discussion

NAFLD is characterized by abundant TG accumulation in the liver. In the present study, we showed that CLW possessed the inhibitory activity of lipid accumulation in hepatocytes. Elevated FFAs from dietary fat intake are considered the crucial risk factor for fatty liver. Frequently, not only patients with NAFLD but also those with obesity and type 2 diabetes show higher FFA levels in serum and in the liver [24]. Therefore, an FFA mixture to induce lipid accumulation in HepG2 cells was used. In previously published studies, oleic acid and palmitic acid led to lipid accumulation in hepatocytes [25,26]. Our study confirmed that FFA exposure increased lipid accumulation. However, cell treatment with CLW and FFA decreased accumulation. These observations were further confirmed by an HF diet-fed animal experiment. The HF diet led to the development of fatty liver, consistent with that seen in a previous study [27], whereas CLW administration lowered the lipid accumulation. Alleviation of lipid accumulation in hepatocytes was more clearly shown by H&E staining. According to the microscopic examination of the CLW-treated mouse liver cells, while being fed the HF diet, the production of lipid droplets was significantly prevented.

Fatty acid uptake factors such as CD36, FATP2, and FATP5 actively function when excessive fatty acids are present owing to high fat intake or obesity, but not in normal conditions. In our study, the HF group remarkably up-regulated the expression of FFA uptake factors, which is in agreement with earlier studies [28]. These results provide evidence that the CLW administration disturbs FFA uptake in the liver by suppressing fatty acid uptake factors.

Despite being useful energy sources, an excess of FFA evokes excessive β-oxidation and eventually produces high ROS levels [29]. Overproduction of ROS could result in the hepatocellular membrane being attacked because of the inactivation of enzymatic and non-enzymatic antioxidants and lipid peroxidation in the liver, followed by liver injuries. When fatty acid uptake is suppressed, the overproduction of ROS could be inhibited. Serum ALT and AST levels are useful indicators of liver damage caused by oxidative stress [30]. Significantly decreased serum enzyme levels were observed in mice co-supplemented with a CLW and HF diet. Moreover, MDA, which is mainly known as a marker of oxidative stress [31], was markedly reduced in the CLW groups. Furthermore, the CLW supplementation ameliorated the impaired antioxidative defense system in mice, as indicated by the restoration of liver antioxidants such as CAT, SOD, GST, GPx, GR, and GSH. These results suggest that CLW has a hepatoprotective potential against oxidative stress.

Oxidative stress is a common risk factor for lipid accumulation in hepatocytes. Due to oxidative stress, alteration in several transcription factors related to TG metabolism causes NAFLD [32]. SREBP-1c is a transcription factor that regulates the synthesis of fatty acids, cholesterol, and triglycerides. It promotes the expressions of ACC and FAS and eventually leads to fatty acid synthesis. SREBP-1c and AMPK are negatively correlated as AMPK activity controls SREBP-1c inhibition [33]. Several studies have shown that FFA-induced HepG2 cells and mice fed an HF-diet suppressed AMPK activity [34,35]. In our study, AMPK expression was remarkably down-regulated by the HF-diet. In addition, as shown by western blotting, the phosphorylation of the AMPK and AMPK ratio was decreased, indicating the inactivation of AMPK. However, supplementation with CLW up-regulated AMPK expression and down-regulated SREBP-1c, FAS, and ACC expressions. 

Expression levels of CPT-1 and PPAR-α, well-known regulators of fatty acid β-oxidation, were significantly increased in the CLW groups. CPT-1 is inhibited by malonyl-CoA, which is produced by ACC [36]. PPAR-α, the main factor for regulating β-oxidation, can potentially prevent NAFLD [37]. Up-regulations of CPT-1 and PPAR-α by supplementation with CLW suggest that CLW contributes to decreasing β-oxidation, resulting in the inhibition of lipid accumulation in hepatocytes.

Silymarin, an approved agent for the treatment of liver damage, was used as a positive control in our study to verify the potential of CLW to produce a comparable hepatoprotective effect. It prevents oxidative stress as well as lipid accumulation [11,38]. In our study, supplementation with CLW showed similar potential for the prevention of NAFLD to silymarin.

NAFLD is one of the most common liver diseases. NAFLD is associated with obesity, diabetes, and dyslipidemia. In general, lipid metabolism and accumulation in hepatocytes leads to hepatic steatosis, the first step of NAFLD. NAFLD can progress to NASH, advanced hepatic fibrosis, and cirrhosis [39,40]. The present study dealt with the steatosis stage of NAFLD in an animal model. We confirmed the potential of CLW to prevent the steatosis. Further study with human subjects is necessary for safe administration with potent efficacy.

## 5. Conclusions

In conclusion, our study is the first to report that treatment with water extract of *C. longa* is effective in the prevention of NAFLD. It is presumed that the mechanism of hepatoprotection by CLW supplementation against lipid accumulation and oxidative stress might be due to the modulation of fatty acid uptake. The knowledge gained from the present study could aid in the development of effective therapeutics to protect from the formation of fatty liver.

## Figures and Tables

**Table 1:** Real-time polymerase chain reaction (RT-PCR) primer sequences.

Gene	Primers	Sequence (5′ to 3′)
CYP2E1	Forward Reverse	5′- CGTGGAAATGGAGAAGGAAA-3′ 5′- GGTGATGAACCGCTGAATCT-3′
AMPK	Forward Reverse	5′- GGCACCCTCCCATTTGATG-3′ 5′- ACACCCCCTCGGATCTTCTT-3′
ACC	Forward Reverse	5′- TGCAGATCTTAGCGGACCAA-3′ 5′- GCCTGCGTTGTACAGAGCAA-3′
CPT-1	Forward Reverse	5′- TGTTGGGTATGCTGTTCATGACA-3′ 5′- GCGGCCTGGGTAGGAAGA-3′
SREBP-1c	Forward Reverse	5′- CGGAACCATCTTGGCAACA-3′ 5′- GCCGGTTGATAGGCAGCTT-3′
PPAR-α	Forward Reverse	5′- AACATCCAAGAGATTTCGCAATC-3′ 5′- CCGTAAAGCCAAAGCTTCCA-3′
CD36	Forward Reverse	5′- TGGAACAGAGGCTGACAACT-3′ 5′- TTGATTTTTGATAGATATGGG-3′
FATP2	Forward Reverse	5′- CTTTCAGCACATTGCTGATTACCT-3′ 5′-CAGTGATCTCAATGGTGTCCTGTAT-3′
FATP5	Forward Reverse	5′- AGCTCCTGCGGTACTTGTGT-3′ 5′- AAGGTCTCCCACACATCAGC-3′
β-Actin	Forward Reverse	5′- ACGGCCAGGTCATCACTATTG-3′ 5′- CAAGAAGGAAGGCTGGAAAAGA-3′
